# Supplementary material for: Hydrodynamic instability at impact interfaces and planetary implications
Source: Nat Commun. 2021 Apr 8;12:2104. doi: 10.1038/s41467-021-22052-z (PMC8032775; doi:10.1038/s41467-021-22052-z)
Supplement: Supplementary file 1 — Supplementary Information [file 41467_2021_22052_MOESM1_ESM.docx]

**Supplementary information**

**Impulsive model approximation:**

We use Richtmyer’s impulsive model^1^ to estimate the size of the mixing zone relative to that of the shocked zone of two impacting bodies, in order to determine:

1. The size of the mixing zone for planetary-scale impacts; and
2. The effect of porosity on the mixing zone.

We consider a simple plane impact of two bodies (Supplementary Fig. 1). Assuming a linear relation between shock velocity *U_s_* and material velocity *U*_p_ for both materials,

(3) *U_s1_* = *C_01_* + *S_1_U_P_*

*U_s2_* = *C_02_* + *S_2_U_P2_*

*U_P2_* = *U_im_ – U_P_*

the shocked-zone growth rate is:

(4) *ΔU_s_* = *U_s1_* + *U_s2_ – U_im_*


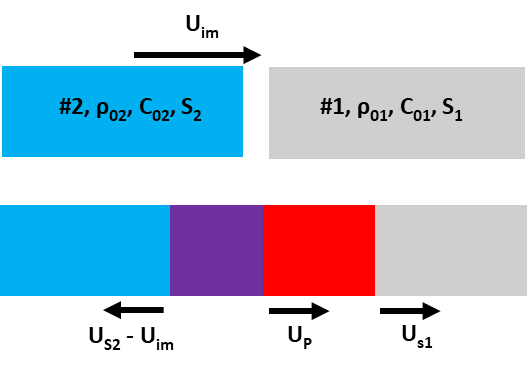


Supplementary Figure 1. **Post impact shock condition.** Top – impact of two bodies (#1 at rest, #2 traveling at *U_im_* to the right) with corresponding density and linear hugoniot parameters (*ρ_0x_*, *C_0x_*, *S_x_*). Bottom – after the impact, the interface is moving with a matching particle velocity (*U_P_*).

We use the Hugoniot relations to determine the growth rate of the shocked zone, with matching particle and shock velocities.2 If one of the surfaces contains a gentle sinusoidal perturbation, as given by eq. (1) in the main text, then an interface instability will develop. Given the analogy between this hydrodynamic instability at impact interfaces and the classical RMI, it is worth comparing our results with Richtmyer’s impulsive model growth rate:

(5) *da*/*dt* = *KU_p_A_t+_a_+_*

Subscript + refers to post-impact or post-shock values; the wavenumber *K* = 2π/*λ*, where *λ* is the perturbation wavelength; *a_+_* is the minimal amplitude just after impact, *a_+_* = *a_0_*(1 – *U_p_*/*U_im_*); and *A_t+_* is the post-shock Atwood number, *A_t+_* = (*ρ_02+_ – ρ_01+_*)/(*ρ_02+_* + *ρ_01+_*).

For the RMI, the shock is initially refracted due to the differences in material properties across the initial interface. However, in the impact case, the post-impact wavy shock structures are generated even for identical materials, purely for geometric reasons. Therefore, we use *A_t+_* = 1 for our impulsive model of the impact instability (thereby eliminating it from our impact-case growth-rate formula). The impulsive model is valid for small perturbations, *a* << *λ*, and is therefore only used for describing growth at early times.

Supplementary Fig. 2 compares the impulsive model for the instability amplitude with the results from Fig. 4a in the main text. We scale the amplitude by *a_+_* and ignore the initial transient.


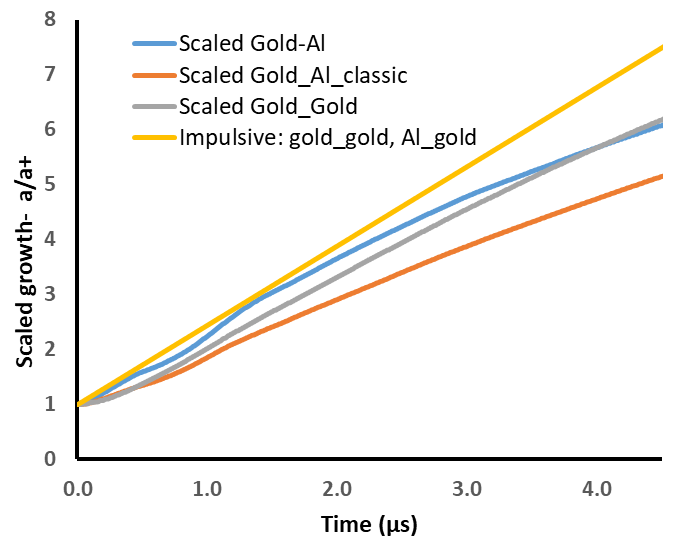


Supplementary Figure 2. **Scaled growth of the instability amplitude.** Al/Au and Au/Au impact instability, modified impulsive model (*da*/*dt* = *KU_p_a_+_*), and Al/Au classical RMI case. In all cases, the matching particle velocity of the Au is 2.3 km/s (equivalent to ~300 GPa)

From this analysis, we find that the amplitude for the impact-induced interface instability is distinctly larger than for the classical RMI. The impulsive model predictions are ~ 20% above the impact simulation results up to ~ 3.5 µs, which corresponds approximately to five times the initial amplitude, *a_+_*. After that, as expected due to extra vorticity production, the simulation growth rates slowly decay and deviate increasingly from those of the impulsive model.

As we obtain a constant growth velocity of the shocked (4) and mixing zone (5), the ratio *V_mix_*/*ΔU*_s_, (*V_mix_* = 2 *da*/*dt*) is kept constant for the impact case (all times post impact), giving an approximate value for the ratio of the mixing zone to the shocked zone volumes (in 3D, lengths in 1D): supplementary table 1.

|  | ***U_impact_***  **[km/s]** | ***V_mix_*/*ΔU_s_***  **[%]** |
| --- | --- | --- |
| ***Al/Gold**** | 9.5 | 8.90 |
| Al/Gold | 30.0 | 17.0 |
| Al/Gold | Analytic limit | 28.6 |
| ***Gold/Gold**** | 4.6 | 8.3 |
| Gold/Gold | 10.0 | 13.3 |
| Gold/Gold | 30.0 | 20.0 |
| Gold/Gold | Analytic limit | 27.5 |
| ***Iron/Dunite**** | 10.5 | 11.3 |
| Iron/Dunite | 30 | 18.5 |
| Iron/Dunite | Analytic limit | 28.2 |

**Supplementary Table 1.** Ratio of mixed-zone to shocked-zone sizes for different impact velocities, based on the impulsive model. Here, *a_0_*/*λ* = 0.05 for all cases, and the examples discussed in the main text are marked by *.

The analytic limits in the supplementary table 1, stand for the limit *V_impact_* 🡪 ∞ as given in (8), below. Under this condition we can neglect the effect of *C_0_* for both impacting materials, such that:

(6) *U_s1_* = *S_1_U_P_*

*U_s2_* = *S_2_U_P2_*

Defining $\delta=1+ \sqrt{\frac{\rho_{01}S_{1}}{\rho_{02}S_{2}}}$ , we get:

(7) *U_p_* = *U_impact_*/*δ*

*ΔUs* = *U_impact_*(*S_1_*/*δ* – *S_2_*/*δ* + *S_2_ –* 1)

(8) $\frac{V_{mix}}{{\Delta U}_{s}}=2a_{0}K\frac{\delta-1}{\delta^{2}(\frac{S_{1}}{\delta}-\frac{S_{2}}{\delta}+S_{2}-1)}$

Thus, for high impact velocity, the ratio *V_mix_*/*ΔU_s_* can reach up to ~30%, depending on material properties (supplementary table 1). The underlying assumption *a*/*λ* << 1 is valid for early times after impact.

**Effect of porosity**

Because small asteroids and moons representative of planetesimals and other bolides in the early Solar System tend to exhibit considerable porosity, we examine how the relative mixing-zone size *V_mix_*/*ΔU_s_* is affected by the porosity of impacting materials. For simplicity, we consider a symmetric impact between identical materials. For an impact velocity *U_impact_*, the particle velocity is half the impact velocity (*U_p_* = *U_impact_*/2), and the post-impact amplitude is also half the initial value, *a_+_* = *a_0_*(1 *– U_p_*/*U_impact_*)]. Therefore, from the impulsive model of the impact instability, the disturbance growth rate (*V_mix_* = 2*da*/*dt*) is identical, regardless of the impacting material, for the same wavelength (*λ*), initial amplitude (*a_0_*) and impact velocity.

Supplementary Fig. 3 gives an example of the *Us-Up* curves for porous media, showing that over a wide range of particle velocities (0-5 km/s), shock velocities are lower in the porous relative to the normal-density material. This implies that growth of the shocked zone (*ΔU_s_* = *U_s1_* + *U_s2_* *– U_impact_*) decreases with increasing porosity.

Therefore, for fixed impact velocity, the ratio of mixed zone to shocked zone increases with increasing porosity of the impacting materials.


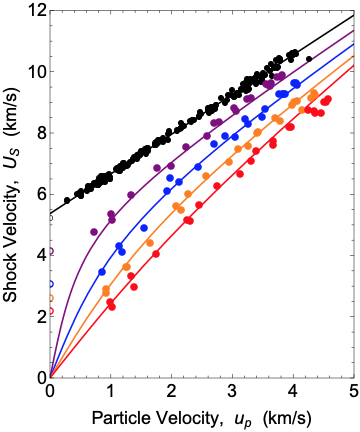


Supplementary Figure 3. **Experimental data for 2024 Al alloy**. Porosities of 0 (black) and approximately 10 (purple), 20 (blue), 30 (orange) and 40 percent (red), with the curves showing the result of a Mie-Grüneisen model.3.^3^

**Figures 2 and 3 in the main text – initial conditions for the simulation:**

To achieve the same post-impact shock pressure in the high-Z material (Au) as for the impact-interface instability with Al impacting at 9.5 km/s, we lower the impact velocity to 9.43 km/s for the classic RMI case (the initial shock wave is generated by an up-range Al-Al impact: Fig. 3). Similarly, to characterize the impact-interface instability, we use an impact velocity of 4.6 km/s for Au/Au and 10.5 km/s for dunite impacting iron in order to achieve comparable peak shock pressures to the case of Al impacting Au.

**Supplementary references**

^1^Richtmyer RD. 1960. Taylor instability in shock acceleration of compressible fluids. Comm. Pure Appl. Math. 8:297–319.

^2^Shalom Eliezer, Heinrich Hora, Ajoy Ghatak, *Fundamentals of Equations of State*, World Scientific Publishing Co Pte Ltd (2002).

^3^R. G. McQueen, S. P. Marsh, J. W. Taylor, J. N. Fritz and W. J. Carter, The equation of state of solids from shock wave studies, in *High Velocity Impact Phenomena*, edited by R. Kinslow, Academic Press, San Diego, CA, pp. 294-419 and 515-568 (1970).
