# Supplementary material for: Hydrodynamic instability at impact interfaces and planetary implications
Source: Nat Commun. 2021 Apr 8;12:2104. doi: 10.1038/s41467-021-22052-z (PMC8032775; doi:10.1038/s41467-021-22052-z)
Supplement: Supplementary file 3 — Description of Additional Supplementary Files [file 41467_2021_22052_MOESM3_ESM.pdf]

Description of Additional Supplementary Files

Title: Supplementary Movie

Description: Au-Au_Impact_Instability

Title: Supplementary Movie

Description: Al-Au_Impact_Instability
